# Supplementary figures and images for: Characterization and Phylodynamics of Reassortant H12Nx Viruses in Northern Eurasia
Source: Microorganisms. 2019 Dec 3;7(12):643. doi: 10.3390/microorganisms7120643 (PMC6956379; doi:10.3390/microorganisms7120643)

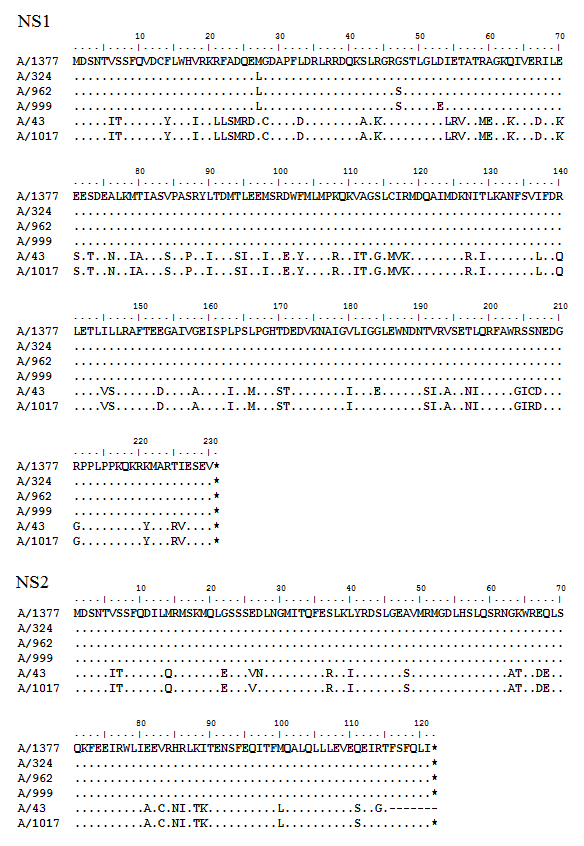

Supplement: Supplementary file 1 [file microorganisms-07-00643-s001.zip › Supplementary/Figure S1.tif]

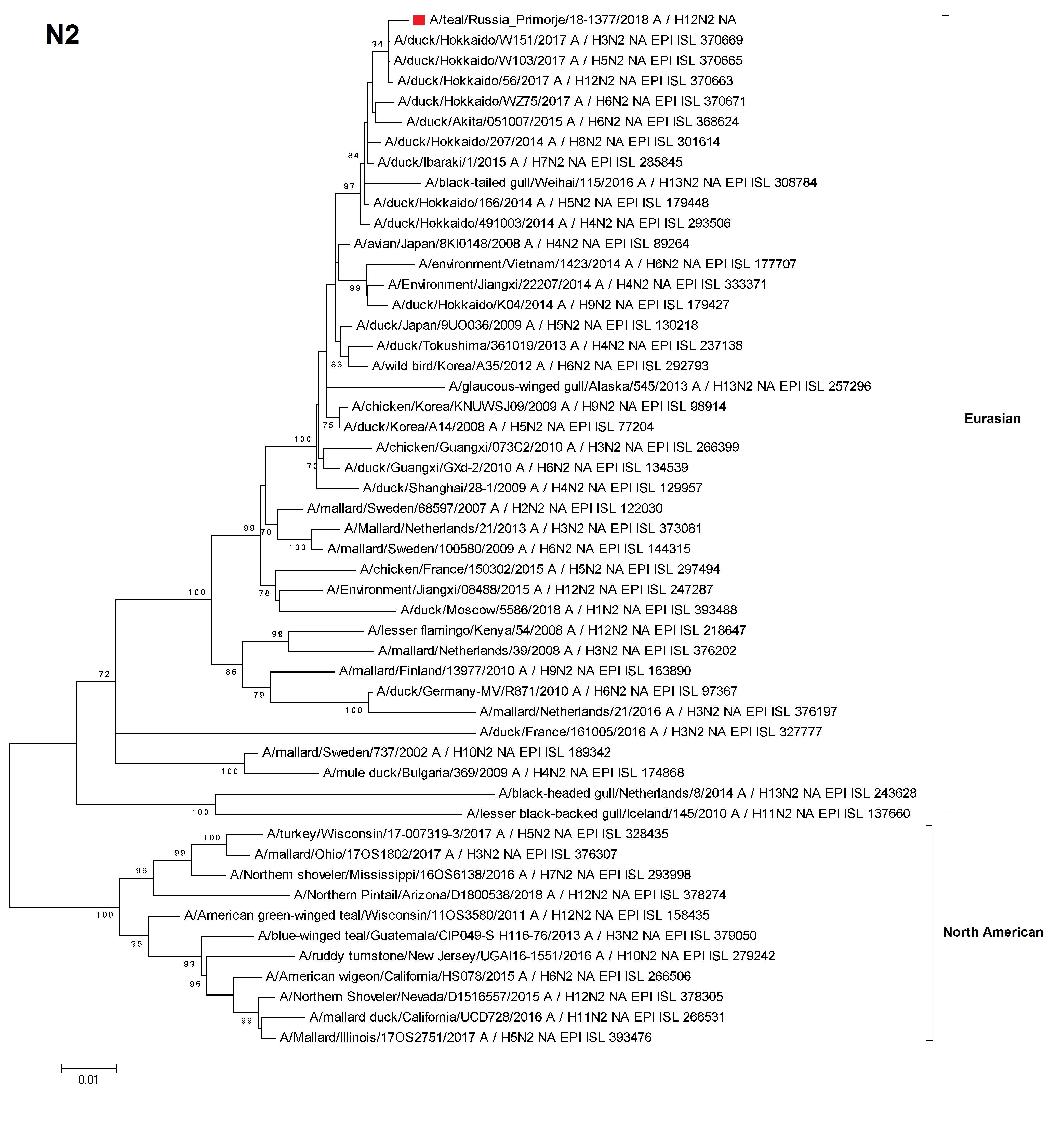

Supplement: Supplementary file 1 [file microorganisms-07-00643-s001.zip › Supplementary/Figure S2.tif]

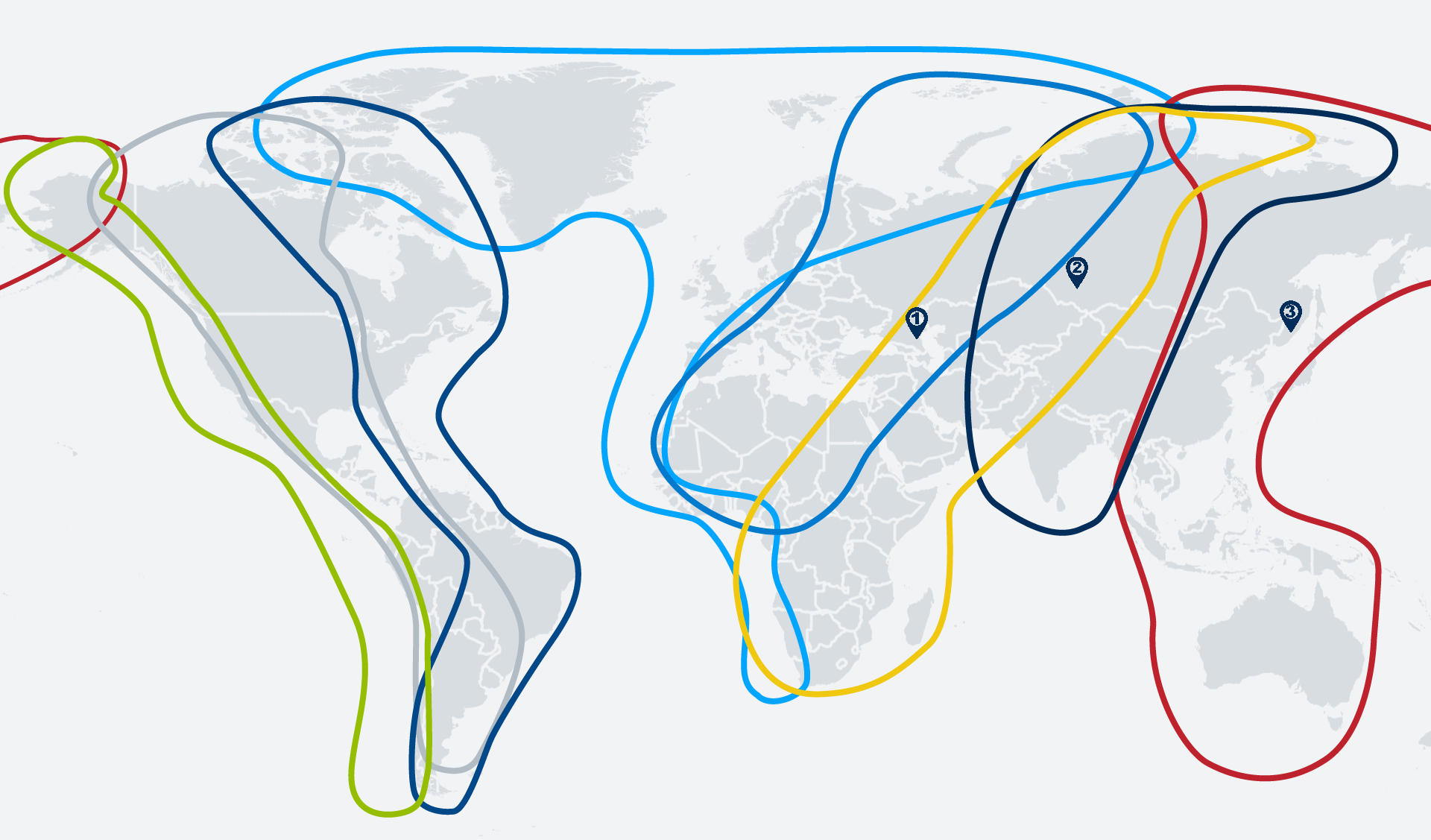

Supplement: Supplementary file 1 [file microorganisms-07-00643-s001.zip › Supplementary/Figure S3.png]

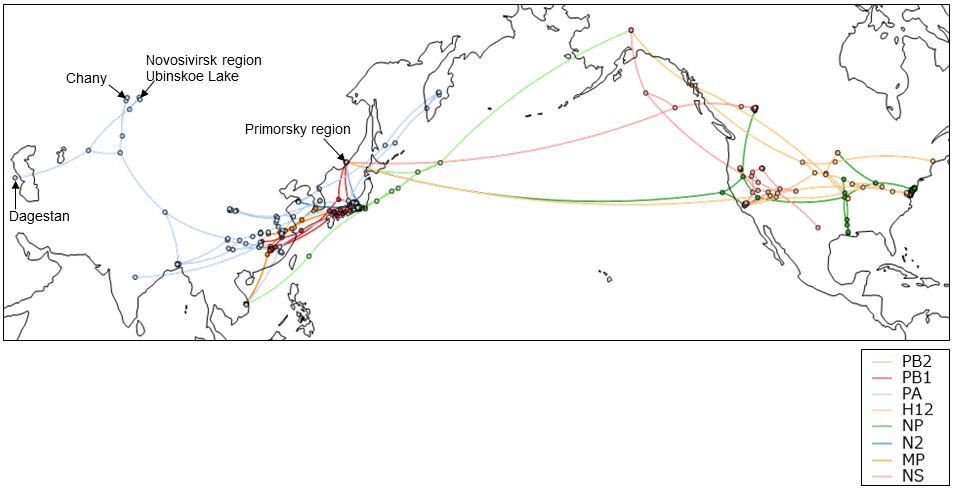

Supplement: Supplementary file 1 [file microorganisms-07-00643-s001.zip › Supplementary/Figure S4a.tif]

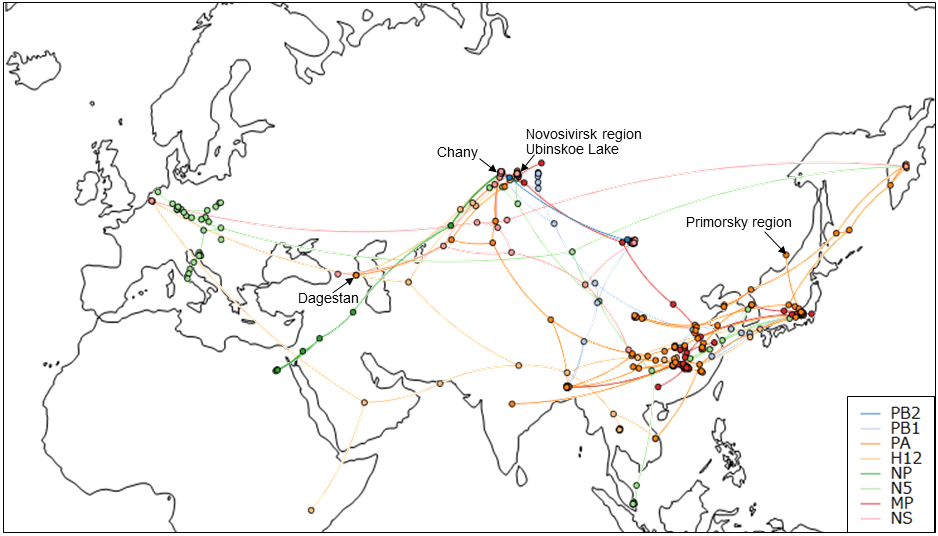

Supplement: Supplementary file 1 [file microorganisms-07-00643-s001.zip › Supplementary/Figure S4b.tif]

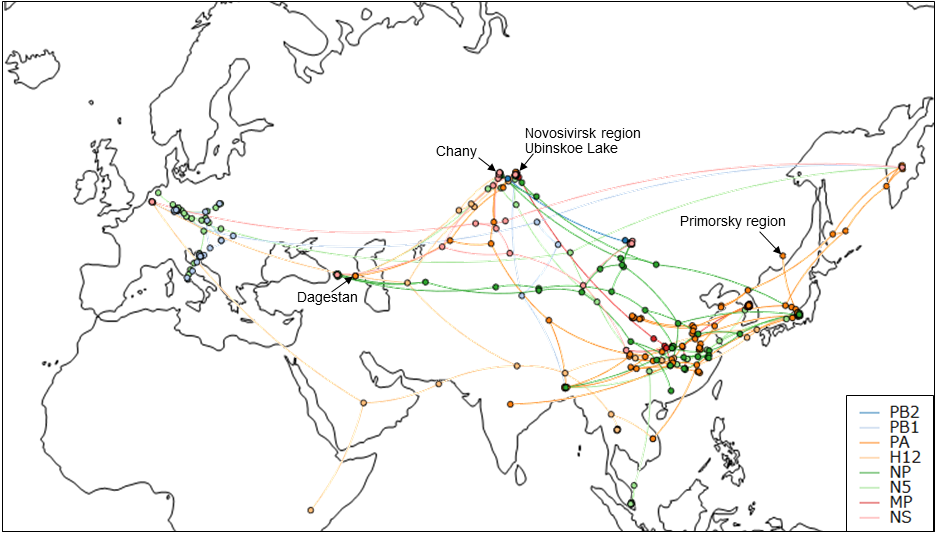

Supplement: Supplementary file 1 [file microorganisms-07-00643-s001.zip › Supplementary/Figure S4c.tif]

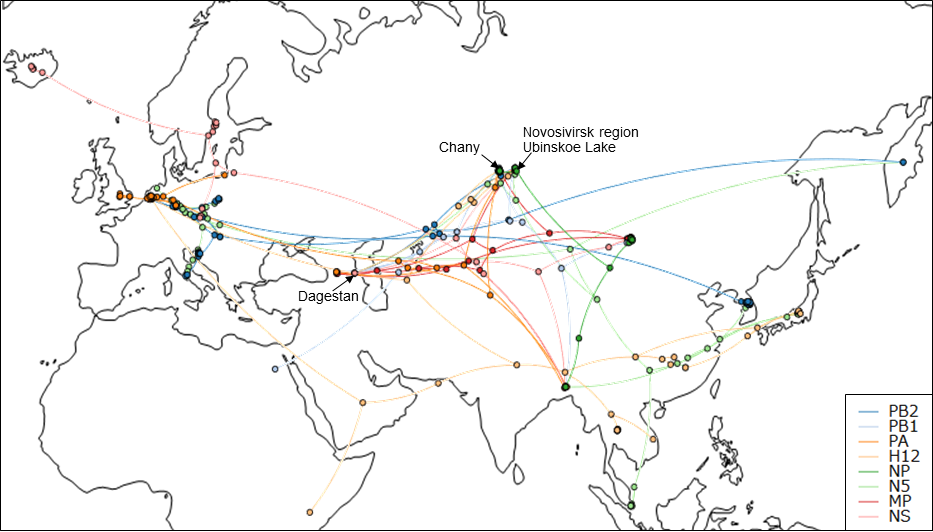

Supplement: Supplementary file 1 [file microorganisms-07-00643-s001.zip › Supplementary/Figure S4d.tif]

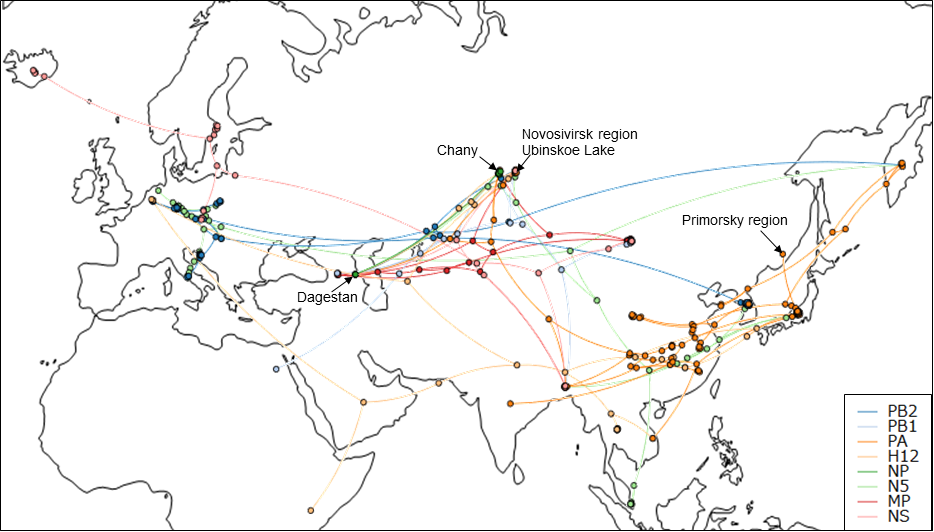

Supplement: Supplementary file 1 [file microorganisms-07-00643-s001.zip › Supplementary/Figure S4e.tif]
